# Physical, psychosocial and dual-career loads as risk factors for injuries and illnesses in elite handball players: a 45-week prospective cohort study

**DOI:** 10.3389/fspor.2025.1664247

**Published:** 2025-08-29

**Authors:** Kristina Drole, Armin Paravlic, Kathrin Steffen, Mojca Doupona

**Affiliations:** ^1^ Faculty of Sport, University of Ljubljana, Ljubljana, Slovenia; ^2^ Faculty of Sports Studies, Masaryk University, Brno, Czechia; ^3^ Oslo Sports Trauma Research Center, Norwegian School of Sport Sciences, Oslo, Norway; ^4^ Norwegian National Unit for Sensory Loss and Mental Health, Oslo University Hospital, Oslo, Norway

**Keywords:** training load, competition load, academic load, work load, student-athlete, health problems, life events, biopsychosocial injury and illness aetiology model

## Abstract

**Introduction:**

While training and competition load are well-documented risk factors for injury, the influence of dual-career loads, life stressors and overall load on both injury and illness remain less clear. The aim of this study was to investigate whether injury/illness occurrence is influenced by the training, competition, academic and work loads, as well as the overall load (sum of academic/work, training and competition loads) and life events in elite male handball players.

**Methods:**

In this 45-week prospective cohort study, 189 elite male handball players weekly reported their load across training, competition, academic, and work domains. We derived an “overall load” variable as the sum of training, competition, academic and work hours. Health problems, including acute non-contact, overuse injuries and illnesses, were recorded using OSTRC-H2-SLO, while psychosocial load was assessed using the LESCA questionnaire. Multivariate logistic regression and non-parametric tests were used to identify risk factors and group differences.

**Results:**

Injured athletes reported significantly higher training (MD = 2.6 h; *p* < 0.001), and overall loads (MD = 2.9 h; *p* = 0.042), but lower academic loads (MD = 2.5 h; *p* = 0.001) than non-injured athletes. Similarly, ill athletes had higher training load (MD = 1.55 h; *p* = 0.026) and competition loads (MD = 0.23 h; *p* < 0.001) but lower academic loads (MD = 2.24 h; *p* = 0.001). Training load emerged as a significant predictor of both injury (OR = 1.33) and illness (OR = 1.23), and competition load strongly predicted illness (OR = 37.00). Academic and work loads were not significant predictors. Higher LESCA total scores were associated with increased injury (*p* = 0.041) and illness risk (*p* = 0.017), while negative scores were associated with increased illness risk (*p* = 0.012).

**Discussion:**

Training and competition loads are key modifiable risk factors for injury and illness, while dual career might serve as a protective factor. While negative life events appear to be associated with illness, the overall volume of life changes—regardless of whether they are positive or negative—emerges as a significant factor in injury risk. Our results support the development of an integrated biopsychosocial model of athlete's health, where sports- and non-sports-related loads, together with life events shape an athlete's vulnerability to injury and illness.

## 1 Introduction

Elite athletes face numerous physical and psychosocial stressors that exert considerable strain on the body's biological systems. While physical demands such as high training volumes, repetitive movements, and player contact are known to increase the risk of health problems (1, 2), mental stressors—such as competitive pressure, academic or work responsibilities, and life events—can compromise an athlete's immune function and further elevate the risk of injury and illness (3–6).

In Slovenia, the structure of elite sport often reflects a hybrid model. While few teams in the 1st Slovenian male handball league have better financial support and lean more towards professional structure (1/4 of teams), it is common for athletes across many sports, including handball, to combine their sports career with academic studies or employment. This stems from the European initiatives such as the EU Guidelines on Dual Careers of Athletes and the Erasmus + Sport programme that have emphasized the importance of enabling athletes to pursue education and employment alongside sport, encouraging member states and sports bodies to develop structured support systems (7). However, the dual career pathway is not only driven by athlete development strategies (e.g., those supported by the EU and Slovenian Olympic Committee's dual career and talent development plan) but also by the financial limitations of clubs and the fact that athletes cannot rely solely on the income from their sports career. As a result, even elite athletes, such as top-league handball players frequently navigate dual careers, balancing the demands of elite handball with university education, part-time or full-time work.

Although the dual-career pathway offers important advantages such as greater long-term security after sports retirement, a safety net in case of unexpected career termination and improved psychological resilience, it also brings challenges that athletes must navigate (8). Players must manage dense training and competition schedules alongside academic deadlines and job responsibilities, often resulting in chronic time pressure, emotional fatigue, and suboptimal recovery (9, 10). In student-athletes, academic stress has been linked to increased injury and illness occurrence (11–14). The Stress and Injury Model (15) suggests that athletes’ appraisal of stressors—and their cognitive and physiological responses—can influence injury risk, especially in those with high trait anxiety or limited coping resources (e.g., social support). While this model is widely cited, much of the evidence stems from general athletic populations and may not fully reflect the demands faced by dual-career athletes competing at a professional level. In this context, the cumulative impact of physical load, academic pressure, and life stressors can further increase the risk of both injury and illness (4, 16).

Although research on training load among elite athletes has grown in recent years (17), studies examining the relationship between different types of loads and health problems remain scarce. For example, one study described training loads and health problems in handball players but did not explore their associations or causal effects (18), while another focused primarily on statistical approaches to analyzing training load data (19). While earlier research has linked stress to injury in broader athletic populations, no studies have comprehensively examined this association within a holistic load framework that accounts for the combined demands of elite sport, life stressors, and dual-career obligations. To address this gap, the present study investigates how physical, psychosocial (life events), and dual-career loads contribute to injury and illness occurrence in elite male Slovenian handball players. We hypothesized that:

1. Greater overall load would be associated with an increased occurrence of injury and illness.
2. Higher academic or occupational demands would be associated with an increased injury/illness occurrence.
3. Injury and illness rates would be higher during periods of higher academic stress compared with periods of lower academic stress.
4. Major life events would be associated with increased injury and illness occurrence.

## 2 Materials and methods

### 2.1 Study design and participants

We designed the study as a prospective cohort study and conducted it in accordance with the Strengthening the Reporting of Observational Studies in Epidemiology (20) guidelines and A CHecklist for statistical Assessment of Medical Papers (21).

The study sample size (*N* = 149) was calculated prospectively for the purpose of this project (22). We invited male handball players from the Slovenian First Men's Handball League (Tier 4: Elite level) (23), and 189 (23.3 ± 4.4 years) responded to participate, all of whom met the inclusion criteria of being male players over 18 years old and actively competing in the league. A total of 33 athletes discontinued participation for various reasons: transfers (*n* = 6), loans (*n* = 5), moving into a goalkeeper coaching role (*n* = 1), and changes of the head coach (*n* = 21). Data from these individuals were included in the analysis until the point of their withdrawal. Eligible players who agreed to participate were informed of the study's purpose and asked to provide written consent. The study was conducted in compliance with the latest version of the Declaration of Helsinki and received approval from the National Medical Ethics Committee of Slovenia (approval number: 0120-109/2022/3). Additionally, the study was prospectively registered on ClinicalTrials.gov (registration number: NCT05471297).

### 2.2 Materials and procedure

We followed the athletes through 45 weeks between July 19th 2022 and June 2nd 2023 during the entire 2022/23 handball season, according to the previously published protocol (22). In collaboration with their support staff—including coaches, strength and conditioning specialists, and physiotherapists—the athletes submitted weekly reports on the following:

(1) Health Problems

Health problems were collected using the Slovenian version of the Oslo Sports Trauma Research Center Questionnaire on Health Problems (OSTRC-H2-SLO) (24). For the purpose of the current study, only first health problem occurrence was taken in account. Furthermore, to assess the predictive value of different types of load on injury and illness occurrence, we excluded contact injuries, as they are typically caused by external forces rather than by training, competition or psychosocial load.

Consequently, health problems were categorized into two distinct categories:

(a) Category 1: Acute non-contact and overuse injuries
(b) Category 2: Illnesses

(2) Load

Load was reported across four categories:

• Training Load: Divided into sport-specific (handball) training and strength and conditioning training.
• Competition Load: Measured by the number of minutes played in games, converted to hours for the purpose of statistical analysis.
• Academic Load: Time spent in lectures, exams, practical courses, and studying. Exam periods were considered as periods of high academic stress, while periods including lectures, practical courses, and studying were classified as pedagogical periods, representing lower academic stress.
• Work Load: Any additional employment undertaken alongside the athletes’ sports careers.

Load calculations were based on a four-week window preceding the injury event. Due to high response rate to the weekly questionnaire, no imputation of missing values was performed.

A composite variable, overall load, was calculated as the sum of training, competition, academic, and work loads, and expressed in hours.

(3) Additionally, the athletes completed the *Life Events Survey for Collegiate Athletes (LESCA)* (4), which is a standardized questionnaire designed to assess significant life events experienced by athletes over the past 12 months. It captures both positive and negative events across personal, academic, and athletic domains, allowing for evaluation of the individual's psychosocial load.

### 2.3 Statistical analysis

We conducted the statistical analysis using the SPSS software (version 29.0, IBM Inc, Chicago, United States of America). Descriptive statistics were used to summarize the outcomes of interest and are presented as mean ± standard deviation, except in Table 1, where they are presented as median ± interquartile range. The normality of data distribution was tested using the Shapiro–Wilk test. Due to the non-normal distribution of most of the data of interest, differences between healthy and unhealthy/injured athletes in load (training load, competition load, academic load, work load, and overall load), and in LESCA derived scores (i.e., negative; positive; and total score) were tested by using Mann–Whitney test. A multivariate binary logistic regression was used to determine whether different load domains represent risk factors associated with the occurrence of health problems. Accordingly, odds ratios (OR) with 95% confidence intervals were calculated and reported.

**Table 1:** Weekly load comparison between non-injured and injured, and between non-ill and ill players.

Weekly load	Category 1: non-injured (*n* = 83)	Category 1: injured (*n* = 81)	Wilcox statistics	Wilcox *p*-value
Training load (hours)	8.77 ± 3.90	10.50 ± 3.62	2,028.00	*p* < 0.001
Competition load (hours)	0.20 ± 0.24	0.25 ± 0.62	3,282.50	0.796
Academic load (hours)	0.19 ± 6.85	0.00 ± 0.00	3,437.00	0.001
Work load (hours)	0.00 ± 3.67	0.00 ± 5.50	2,893.00	0.566
Overall load (hours)	12.11 ± 9.86	13.31 ± 11.81	2,744.00	0.042
Weekly load	Category 2: non-ill (*n* = 146)	Category 2: ill (*N* = 39)	Wilcox statistics	Wilcox *p*-value
Training load (hours)	9.05 ± 4.09	10.00 ± 4.44	2,187.00	0.026
Competition load (hours)	0.20 ± 0.21	0.44 ± 0.43	1,849.00	*p* < 0.001
Academic load (hours)	0.00 ± 6.88	0.00 ± 0.25	2,910.50	0.032
Work load (hours)	0.00 ± 5.18	0.00 ± 0.00	2,618.00	0.213
Overall load (hours)	11.84 ± 9.28	12.52 ± 7.53	2,506.00	0.252

Data presented as median ± interquartile range.

To assess differences in the occurrence of health problems between athletes with or without dual career and periods of high and low academic stress a Pearson's Chi-squared test was conducted. Additionally, Spearman's rank correlation was used to examine the associations between the LESCA negative score, LESCA total score, and the number of all health problems, injuries, and illnesses.

The following thresholds of the correlation coefficient were used to assess magnitude of the relationships analysed: weak ≤0.35; 0.36 ≤moderate <0.67; 0.68 ≤high <1 (25). Statistical significance for all analyses was accepted at *p* ≤ 0.05.

## 3 Results

Detailed characteristics of the study sample, including age, training experience, weekly training and competition loads, as well as academic and occupational engagement, have been described previously (26). This earlier publication also presents the prevalence and types of reported acute, overuse injuries and illnesses in this cohort. Briefly, athletes accumulated 50,778 h of handball-related activity, comprising 3,675 h in matches and 47,103 h in handball training. Additionally, they have dedicated 20,674 h to strength and conditioning training. Across the study period, 316 health problems were recorded, leading to a total of 3,318 days of absence, with an average of 10.7 days lost per health problem. The most commonly reported health problems were acute lower limb injuries and overuse injuries affecting the knee, lower back/pelvis and shoulders. Infections, particularly upper respiratory tract infections, represented the majority of illness cases. The weekly load comparison between non-injured and injured, and between non-ill and ill players is presented in Table 1.

### 3.1 Physical load

#### 3.1.1 Acute non-contact and overuse injuries

Results showed that injured athletes (*n* = 81) had significantly higher training load [mean difference (MD) = 2.6 h; *p* < 0.001], higher overall load (MD = 2.9 h; *p* = 0.042), and lower academic load (MD = 2.5 h; *p* = 0.001) than their non-injured counterparts. Differences in competition load and work load were also observed, but they did not reach statistical significance (*p* > 0.05 for both).

#### 3.1.2 Illnesses

Results showed that athletes who reported an illness (*n* = 39) had significantly higher training load [mean difference (MD) = 1.55 h; *p* = 0.026], higher competition load (MD = 0.23 h; *p* < 0.001), and lower academic load (MD = 2.24 h; *p* = 0.001) than their healthy counterparts. Differences were also noted in work load and overall load, but these did not reach statistical significance (*p* > 0.05 for both).

### 3.2 Dual-career load

#### 3.2.1 Acute non-contact and overuse injuries

Results indicated that dual-career athletes (*n* = 106) were no more likely to sustain an injury than athletes devoted solely to sport (OR = 0.83; 95% CI 0.61–1.14; *χ*² = 1.13; *p* = 0.288). Likewise, when injury odds were compared across academic periods, dual-career athletes showed identical injury risk in exam and pedagogical periods (OR = 1.01; 95% CI 0.51–1.86; *χ*² = 2.32; *p* = 0.999).

#### 3.2.2 Illnesses

Results indicated that dual-career athletes (*n* = 106) were no more likely to report illnesses than athletes devoted solely to sport (OR = 0.63; 95% CI 0.37–1.14; *χ*² = 1.05; *p* = 0.098). Likewise, when illnesses odds were compared across academic periods, dual-career athletes showed no difference in illnesses risk in exam and pedagogical periods (OR = 0.43; 95% CI 0.06–1.49; *χ*² = 0.99; *p* = 0.318).

### 3.3 Load as risk factor for injury and illness

#### 3.3.1 Acute non-contact and overuse injuries

Results from multivariate binary logistic regression revealed that training load was the only significant risk factor for injury occurrence: each hour of increase in training load was associated with a 33% increase in the odds of injury (OR = 1.33, 95% CI 1.17–1.55, *β* = 0.29, *z* = 3.99, *p* < .001). Competition load showed a positive but non-significant association (OR = 2.65, 95% CI 0.77–9.66, *β* = 0.97, *z* = 1.53, *p* = 0.127). Average academic load was negatively but non-significantly related to injury risk (OR = 0.94, 95% CI 0.88–1.01, *β* = –0.06, *z* = –1.70, *p* = 0.089), as was work load (OR = 1.01, 95% CI 0.98–1.04, *β* = 0.009, *z* = 0.55, *p* = 0.581).

#### 3.3.2 Illnesses

Results of the multivariate binary logistic regression revealed that higher training load was significantly associated with greater illness risk: each additional hour of training increased the odds of illness by 23% (OR = 1.23, 95% CI 1.04–1.50; *β* = 0.21; *z* = 2.23; *p* = 0.026). Competition load displayed an even stronger positive association (OR = 37.00, 95% CI 7.71–222.68; *β* = 3.61; *z* = 4.25; *p* < 0.001). In contrast, academic load (OR = 0.95, 95% CI 0.84–1.04; *β* = –0.05; *z* = –1.03; *p* = 0.305) and work load (OR = 0.99, 95% CI 0.95–1.03; *β* = –0.01; *z* = –0.45; *p* = 0.656) were not significantly related to illness occurrence.

### 3.4 Psychosocial load

When comparing athletes who sustained injuries to those who did not, a statistically significant difference was observed for LESCA total scores (Injured vs. non-injured: 7.94 ± 17.54 vs. 3.57 ± 9.56; *U* = 3,993, *Z* = 2.047, *p* = 0.041). However, LESCA positive scores (*U* = 3,556, *Z* = 0.541, *p* = 0.589) and LESCA negative scores (*U* = 2,910, *Z* = −1.942, *p* = 0.051) showed no statistically significant differences.

In comparisons between athletes who reported illness and those who did not, statistically significant differences emerged for LESCA negative scores (Ill vs. healthy: −5.77 ± 10.06 vs. −2.40 ± 7.10; *U* = 2,497.5, *Z* = −2.506, *p* = 0.012) and LESCA total scores (Ill vs. healthy: 9.53 ± 17.04 vs. 6.0 ± 15.58; *U* = 3,769.5, *Z* = 2.396, *p* = 0.017). LESCA positive scores (*U* = 3,346.5, *Z* = 0.931, *p* = 0.352) did not differ significantly between these groups.

Weak to moderate associations were observed between LESCA scores and number of health problems. Specifically, for the total number of health problems, LESCA negative scores showed a moderate association (*r* = 0.36, *p* < 0.001), while LESCA total scores showed a weak association (*r* = 0.33, *p* < 0.001). Similarly, weak associations were found between LESCA scores and the total number of injuries: LESCA negative scores (*r* = 0.33, *p* < 0.001) and LESCA total scores (*r* = 0.30, *p* < 0.001). Finally, for the total number of illnesses, LESCA negative scores (*r* = 0.20, *p* = 0.005) and LESCA total scores (*r* = 0.19, *p* = 0.007) also demonstrated weak associations.

## 4 Discussion

The aim of this study was to investigate the relationship between various domains of load—physical (training and competition), dual career (academic/work), and overall load—and the occurrence of injuries and illnesses in elite male handball players. Additionally, the study explored the role of psychosocial stressors, measured by the LESCA questionnaire, and health problems in athletes.

Our findings support the assumption that greater training and overall load is associated with increased injury risk, while higher training and competition load is associated with increased illness risk. Notably, the regression analysis highlighted training load as a key risk factor: each additional hour of training was associated with a 33% increase in injury odds and a 23% increase in illness odds.

### 4.1 Physical load and health problems

These results support the fact that excessive physical stress can lead to fatigue, impair recovery and body's repair mechanisms and subsequently increase susceptibility to injuries (1, 27). Without sufficient recovery between training sessions, microtrauma can accumulate, ultimately increasing injury risk. Moreover, high training loads can also affect immune function by altering immune efficiency. At rest, trained athletes often show lower circulating leukocyte counts compared to non-athletes (28, 29), while repeated elevation of stress hormones (especially glucocorticoids) caused by ongoing intense exercise can induce cumulative immunosuppression (30). When recovery is insufficient, athletes may enter a prolonged “open window” of reduced immunity, increasing their vulnerability to illness (31).

Competition load showed a strong and significant association with illness occurrence, likely due to the elevated physiological and psychological demands of matches. Match play involves greater emotional arousal, intensity, physical contact and exposure to airborne pathogens compared to training—all of which may compromise immune function (5). Physiological stress from competition triggers immune responses similar to those seen in infections — such as elevated circulating leukocytes, with these effects being dependent on exercise intensity and duration (32). In our cohort, the majority of reported illnesses were indeed viral infections (26), further supporting this physiological explanation and previous findings (33). In team sports like handball, where close physical contact is unavoidable during matches, this transient immune suppression, combined with increased pathogen exposure, likely accounts for the observed strong association between higher competition load and illness occurrence.

### 4.2 Dual-career load and health problems

Interestingly, our results did not support the assumption that athletes with greater academic or work-related commitments face higher risks of injury or illness. Dual-career athletes did not report higher health problem rates than their peers who focus solely on sport, and no significant differences were observed across academic periods such as exam or pedagogical phases. This finding challenges the commonly held assumption that dual-career athletes are particularly vulnerable due to cumulative stress and time pressure (9, 10). One possible explanation lies in the protective role of structured dual-career pathways, where engagement in academics may offer psychological distance from the demands of sport, enhance identity development, resilience, coping strategies, life balance and reduce emotional overinvestment in athletic success (34–36). It is possible that athletes who successfully balance dual careers have developed superior self-regulation, time management, and coping strategies, buffering the impact of stress on their physical health (37). Furthermore, athletes investing less time in academic activities have more capacity to increase physical training, potentially leading to overreaching or insufficient recovery.

While Slovenian handball players in this study appeared to manage their sport and non-sport demands effectively, showing no increased risk of health problems related to dual-career engagement, further comparative data from similar elite handball populations across Europe are needed to contextualize these findings more broadly. Interestingly, our results contrast with findings from previous studies on student-athletes in other countries. For example, Hamlin and colleagues (11) reported increased injury risk during academic exam periods among New Zealand student-athletes from various sports, and similar trends were observed in U.S. collegiate football athletes (13), where periods of high academic stress were linked to elevated injury and illness risk. However, methodological differences between studies limit the extent to which direct comparisons can be made. Unlike our study, which implemented weekly self-reported surveillance and recorded 316 health problems over one competitive season (26), Hamlin and colleagues relied on injury and illness reports from medical staff, potentially missing subclinical or unreported cases. As a result, only 259 health problems were recorded across four years. These differences underline the importance of standardized surveillance methods in sports injury research and highlight the uniqueness of our study in capturing a more complete picture of athlete health within a dual-career handball context.

### 4.3 Psychosocial load and health problems

Our findings indicate that injured athletes had significantly higher total LESCA scores than non-injured athletes, indicating a potential association between cumulative life stress and injury risk. Although the effect size may be modest, this finding aligns with previous research suggesting that elevated life stress can increase the likelihood of injuries, possibly through mechanisms such as attentional disruption, muscle tension, or compromised immune function (12, 30). The role of psychosocial factors in injury risk has been highlighted in models such as Andersen and Williams' Stress–Injury Model (15), which identifies key psychosocial predictors: personality, history of stressors, and coping resources. Among these, perceived stress—especially from negative life events—has been identified as the most robust predictor (3). More specifically, major (e.g., negative life event stress) as well as minor (e.g., daily hassles) negative life events have been found to increase the likelihood of becoming injured among football players (1) and female team-sport players (38). Our results similarly suggest that not only major negative events but the total burden of life stress contributes to athlete's injury risk.

Athletes who reported illness exhibited significantly higher negative LESCA scores and total scores than those who remained healthy, indicating that negative life events, in particular, may contribute to vulnerability to illness. This finding is consistent with psychoneuroimmunological theories, which propose that chronic or intense negative stressors can impair immune system function and increase susceptibility to infections or other illness-related outcomes (12, 30, 32).

### 4.4 Conceptual model of different aspects of load as risk factors for injury and illness

**Figure 1:**
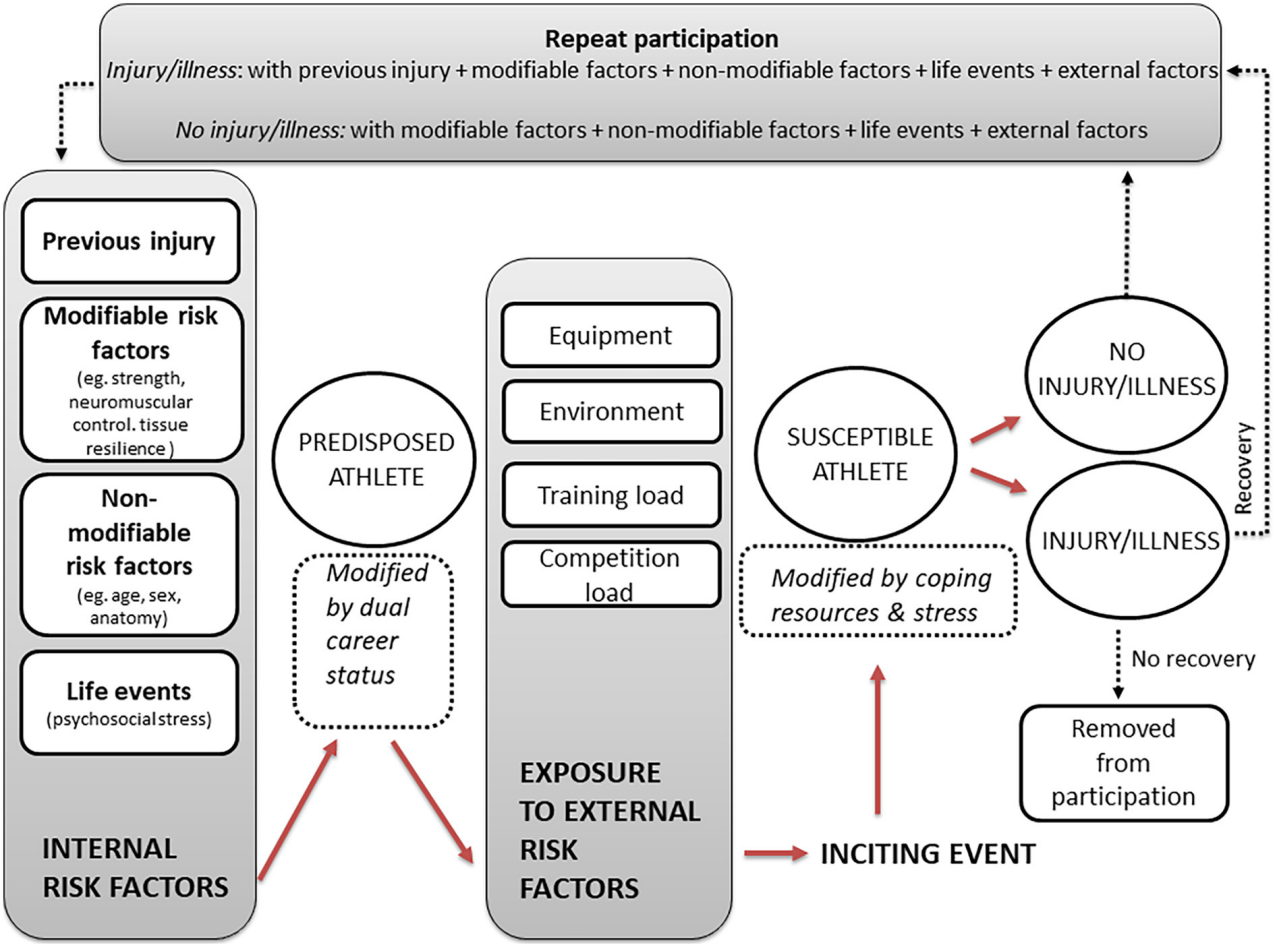
The biopsychosocial injury and illness aetiology model.

The results of the present study allow for the update of Meeuwisse and colleagues' Dynamic, recursive athletic injury aetiology model (39) and The workload—injury aetiology model (27) in terms of how various forms of load (physical, dual-career, psychosocial) influence injury and illness risk in athletes (Figure 1). Compared to previous models, this version incorporates dual-career and psychosocial factors not traditionally emphasized, providing a more holistic understanding of athlete's susceptibility to injury and illness.

At the core of the model are individual modifiable and non-modifiable *predisposing internal risk factors* (such as previous injury, neuromuscular control, strength, age and sex), which shape the athlete's baseline susceptibility. In addition, *psychosocial stress*, operationalized via the occurrence of negative life events emerged as a weak to moderate predictor of injury and illness.

Exposure to *external risk factors* in the form of equipment, environment and load plays a central role in determining whether an athlete transitions from a “predisposed” to a “susceptible” state. In particular:

• *Training load* was a significant predictor of both injury and illness.
• *Competition load* showed a strong relationship with illness risk although it was not significant for injury.
• In contrast, *academic load* and *work load* were not associated with greater risk, and in fact, academic load showed trends toward a protective effect, therefore it was placed as a moderating factor in the model.

After an inciting event, the model includes feedback loops: participation in sport can either lead to injury/illness, triggering recovery and return to participation or removal from participation.

From an applied perspective, this model underscores the effects of physical, dual-career, and psychosocial factors in shaping athlete health outcomes and highlights the importance of balanced load management and psychosocial support in injury prevention strategies. While physical load—particularly training and competition—emerged as the most robust predictor of injury and illness, this does not negate the importance of understanding each athlete's full context. Mismanagement of load not only increases the risk of health problems but also contributes to long-term pain, scholarship loss, and psychological distress at the individual level (40). At the organizational level, injuries result in poorer team performance and higher financial costs for clubs and health systems (41).

Both injury and illness appear to be influenced by the presence, type and number of life stressors. These insights highlight the value of individualized training planning, integrated medical support, and psychosocial resources to help athletes maintain health and performance across both sporting and academic domains. Integrating life stress assessments into athlete monitoring may help identify individuals at greater risk for health problems and inform more holistic prevention strategies that might reduce injury and illness occurrence in athletes.

### 4.5 Limitations and future directions

This study has several important strengths that contribute to the understanding of how different loads influence injury and illness risk in elite handball players. One of the primary strengths lies in the comprehensive monitoring of physical, dual-career and psychosocial loads across an entire competitive season. Unlike many studies that focus solely on training or competition loads, this research includes academic and work loads, alongside with life events, which provides a more holistic picture of athletes' total burden. The inclusion of dual-career loads is particularly relevant in contexts like Slovenia, where many elite athletes must manage academic or employment responsibilities alongside their sports career. Moreover, the weekly self-reporting design enabled a consistent and longitudinal collection of data.

However, several limitations should be acknowledged. First, due to the sample including only athletes from the top Slovenian handball league, this might affect the generalizability of findings to broader athletic populations, including female athletes, youth players, or those from different sports or cultural settings. Second, all load variables and health problems were self-reported using weekly questionnaires. While this approach allows for consistent longitudinal monitoring, it is prone to recall bias and dishonest reporting. Objective methods, such as GPS tracking or heart rate monitoring would improve the accuracy of load assessment. Moreover, the composite variable—overall load was calculated as the unweighted sum of hours across training, competition, academic, and occupational activities. This assumes equal impact of different load types, which may not accurately reflect their distinct physiological and psychological effects.

To address these limitations, future studies should aim to include more diverse samples, incorporating female athletes, youth players, and international comparisons to explore how different cultural and structural factors shape dual-career demands and health. Future research should explore potential mediators and moderators of the stress-health relationship, such as personality traits, coping mechanisms, and training load, as well as the timing and perceived controllability of stressors. A long-term prospectively designed studies would also be valuable to assess causality and the relationship between life stress and health outcomes over numerous seasons.

## 5 Conclusion

Building on existing dynamic models of injury risk, our findings suggest that in elite handball athletes, training and competition loads are key modifiable risk factors for both injury and illness. Moreover, dual-career loads do not contribute to greater injury/illness risk but may rather, through enhanced psychosocial balance, represent a protective factor. Both the quantity and emotional tone of life events contribute to athlete health risks. While negative life events appear to be associated with illness, the overall volume of life changes—regardless of whether they are positive or negative—emerges as a significant factor in injury risk. This underscores the importance of monitoring not only physical loads but also the broader psychosocial context in which athletes live and perform. Our results support the development of an integrated biopsychosocial model of athlete's health, where sports- and non-sports-related loads, together with life events shape an athlete's vulnerability to injury and illness.

## Data Availability

The raw data supporting the conclusions of this article will be made available by the authors, without undue reservation.
